# First Manic Episode With Psychotic Features in a Young Woman as the Initial Presentation of Graves’ Disease: A Case Report

**DOI:** 10.7759/cureus.111461

**Published:** 2026-06-25

**Authors:** Mohamed Azaouagh, Salah-Eddine El Jabiry, Ismail Moumni, Imane Ramdani, Fatima El Ghazouani, Bouchra Oneib

**Affiliations:** 1 Department of Psychiatry, Mohammed VI University Hospital, Oujda, MAR; 2 Maternal-Child and Mental Health Research Laboratory, Faculty of Medicine and Pharmacy of Oujda, Mohammed First University, Oujda, MAR

**Keywords:** bipolar patients, case report, graves’ disease, hyperthyroidism, secondary mania

## Abstract

Graves’ disease can manifest with psychiatric symptoms, notably manic symptoms. We report the case of a 31-year-old patient with no notable medical or psychiatric history, admitted for a first manic episode characterized by a labile mood, psychomotor agitation, and insomnia without fatigue, associated with delusional ideas. Clinical examination found exophthalmos with anterior neck swelling. Biological assessment showed hyperthyroidism with suppressed thyroid-stimulating hormone (TSH), elevated free thyroxine, and positive anti-TSH receptor antibodies, leading to the diagnosis of Graves’ disease. The patient benefited from treatment combining olanzapine, carbamazepine, methimazole, and propranolol. Clinical evolution was marked by rapid improvement of psychiatric symptoms. This case underlines the importance of performing a thorough clinical examination and excluding organic causes before concluding a psychiatric disorder.

## Introduction

Graves’ disease is an autoimmune thyroid disorder that presents with hyperthyroidism. Its prevalence is less than 1%, with a female predominance [1]. Women have an estimated lifetime risk of about 3%, approximately eight times higher than that in men [2]. Graves’ disease is characterized by three main features: diffuse goiter (a uniform, smooth enlargement of the thyroid gland, often accompanied by an audible bruit), Graves’ ophthalmopathy (prominent eyes, eyelid retraction, dry eyes, and diplopia), and pretibial myxedema (thickened, reddish skin over the shins with an orange-peel appearance) [3].

Hyperthyroidism is the clinical syndrome caused by excess circulating free thyroxine (FT4) or free triiodothyronine, or both [4]. In Graves’ disease, hyperthyroidism can lead to psychiatric manifestations such as insomnia, anxiety, and memory and concentration difficulties, as well as somatic symptoms including weight loss, asthenia, tremors, and palpitations [5,6]. In rare cases, patients may present with mania [7].

Mania is defined as a distinct period of abnormally and persistently elevated, expansive, or irritable mood and abnormally and persistently increased activity or energy, lasting at least one week (or any duration if hospitalization is necessary). According to the Diagnostic and Statistical Manual of Mental Disorders, Fifth Edition (DSM-5), the criteria for a manic episode [8], Criterion A requires such a mood disturbance lasting at least one week. Criterion B includes three (or more) of the following symptoms (four if mood is only irritable): inflated self-esteem or grandiosity, decreased need for sleep, more talkative than usual or pressure to keep talking, flight of ideas or subjective experience of racing thoughts, distractibility, increased goal-directed activity or psychomotor agitation, and excessive involvement in activities with a high potential for painful consequences. Criterion C states that the disturbance is sufficiently severe to cause marked impairment in social or occupational functioning, necessitate hospitalization, or include psychotic features. Criterion D specifies that the episode is not attributable to the physiological effects of a substance or another medical condition [8].

Secondary mania is a recognized presentation of bipolar disorder. A meta-analysis of 18 studies reported an overall prevalence of 6.4% for mania in patients with hyperthyroidism [9]. We report a case of a patient admitted for a first manic episode revealing underlying hyperthyroidism. The case is presented in accordance with CARE recommendations [10].

## Case presentation

A 31-year-old married woman, a housewife, with no significant medical history and no family or personal history of psychiatric disorders, presented to the psychiatric department with psychomotor agitation. There was no history of trauma or substance use. The patient lived with her husband, and their marital relationship was described as good and supportive prior to the onset of symptoms. She came from a middle-class background and was the third of five siblings. She had a university education. Her psychomotor development was normal. No premorbid personality traits were reported by the family.

According to her husband, the onset of symptoms dated back to one week prior to admission, with the appearance of insomnia without fatigue, accompanied by logorrhea, disinhibition, and delusional ideas of grandeur, complicated by clastic episodes and hetero-aggression, particularly toward her loved ones. He further described progressively severe insomnia, with the patient sleeping only two hours per night and exhibiting abnormally high energy levels during the day. She began performing household chores excessively, spending time in the garden and working there incessantly. This hyperactivity was accompanied by intense logorrhea, with profuse and continuous speech and abrupt topic switching. Behavioral disinhibition was also present, manifested by obscene and incongruous remarks. Delusions of grandeur emerged, including claims of being the “beauty queen of Morocco” and the “best cook in Morocco.” Financially, excessive and reckless spending was reported: she spent nearly $1,000 in one week, purchasing large quantities of goods at the supermarket and distributing them to neighbors without hesitation.

The clinical picture was complicated by episodes of destructive behavior. During the first episode, the patient smashed the television, a table, and a cupboard. During the second episode, she broke several kitchen utensils (glasses and plates), the kitchen door, and a window, in a context of marked irritability. Physical aggression toward her husband was reported after she struck him when he entered the house without removing his shoes, triggering an acute rage episode. Verbal aggression toward her mother-in-law was also noted during a telephone call.

On admission, the patient was agitated but conscious and fully oriented in time and space. She was wearing makeup and had appropriate, clean attire. Attention was reduced, with marked distractibility. Thought processes were accelerated. Speech was rapid and excessive, with logorrhea and tachyphrenia. Mood was labile, with rapid and unexplained shifts from laughter to tears.

Regarding thought content, several delusional ideas were present: (1) delusion of filiation (the patient claimed to be the daughter of a king); (2) delusion of possession (the patient believed she was inhabited by a devil); (3) religious delusion (the patient claimed to be sent by God); and (4) mood-congruent persecutory ideas (the patient believed people were watching her because of her beauty). She also verbalized multiple plans, including opening a restaurant, building a castle, and opening a supermarket. The patient also had persistent insomnia without fatigue.

On admission, vital signs were as follows: heart rate, 105 beats per minute; blood pressure, 120/80 mm Hg; oxygen saturation, 96% on room air; respiratory rate, 18 breaths per minute; and body temperature, 37.2 °C. The patient was hospitalized and started on treatment. Olanzapine was progressively increased to 15 mg/day (after gradual dose escalation) to treat psychotic symptoms and stabilize mood. Carbamazepine was also initiated and increased to 400 mg/day (after gradual dose escalation). In addition, chlorpromazine was introduced at 200 mg/day for its sedative effects. Alprazolam was administered in a tapering regimen to manage anxiety and agitation.

Specifically, olanzapine was given orally at 10 mg once daily in the evening for the first 10 days, then increased to 15 mg once daily in the evening. Carbamazepine was prescribed orally at 200 mg once daily in the morning for five days, then increased to 400 mg once daily in the morning. Chlorpromazine was administered orally at 100 mg twice daily (morning and evening) for 15 days, then reduced to 100 mg once daily in the evening for six days, then further reduced to 50 mg once daily in the evening for seven days before discontinuation. Alprazolam was given orally at 0.5 mg twice daily (morning and evening) for seven days, then reduced to 0.75 mg/day (half a tablet in the morning and one tablet in the evening) for seven days, then 0.5 mg once daily in the evening for seven days, and finally 0.25 mg once daily in the evening for seven days before discontinuation.

**Figure 1:**
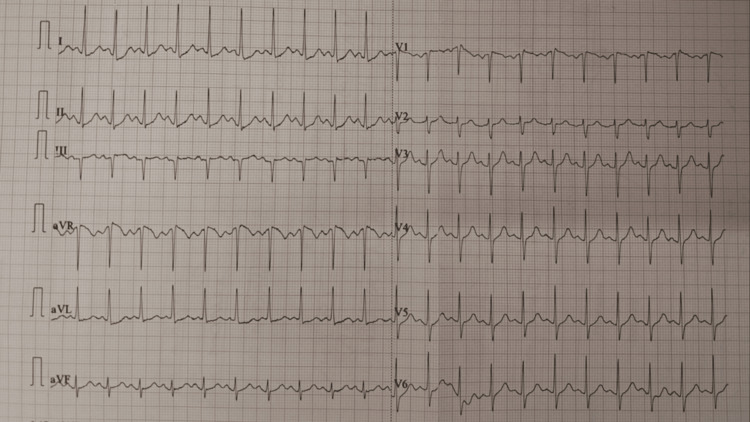
ECG showing regular sinus tachycardia

A complete somatic examination was performed after stabilization of agitation. On inspection, bilateral exophthalmos was noted (the patient reported onset six months prior to admission), along with anterior neck swelling. On palpation, both thyroid lobes were slightly enlarged, soft, and non-tender, with no palpable nodules. No psychometric testing was conducted during hospitalization. The patient underwent an ECG, which showed sinus tachycardia with a regular rhythm (Figure 1).

The biological assessment revealed hyperthyroidism with a low thyroid-stimulating hormone (TSH) level of 0.01 mIU/L (reference: 0.4-4.0 mIU/L) and an elevated FT4 level of 37.33 pmol/L (reference: 9-22 pmol/L), consistent with thyroid dysfunction. In addition, anti-TSH receptor antibodies (anti-TSH receptor antibodies) were positive with a titer >50, and anti-thyroid peroxidase (TPO) antibodies were elevated at 396.61 IU/mL (reference: <34 IU/mL) (Table 1).

**Table 1:** Laboratory findings, reference ranges, and clinical interpretation FT4, free thyroxine; TPO, thyroid peroxidase; TSH, thyroid-stimulating hormone

Parameter	Patient value	Reference range	Interpretation
TSH	0.01 mIU/L	0.4-4.0 mIU/L	Decreased
FT4	37.33 pmol/L	9-22 pmol/L	Elevated
Anti-TSH receptor antibodies	>50	Negative	Positive
Anti-TPO antibodies	396.61 IU/mL	<34 IU/mL	Elevated

**Figure 2:**
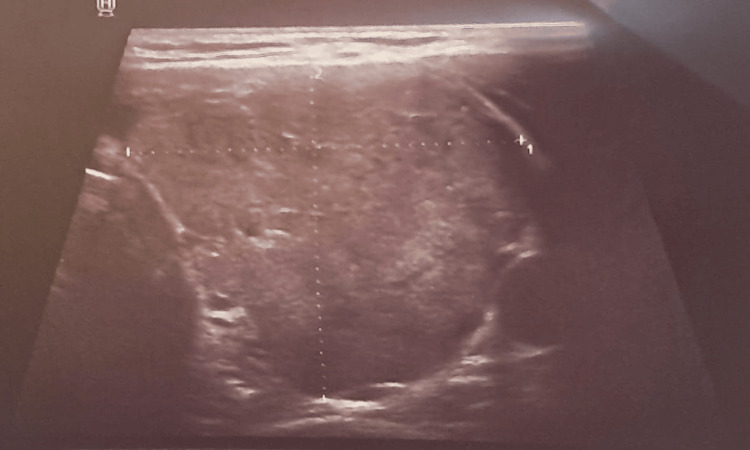
Ultrasound image of the left thyroid lobe showing a goiter involving the left thyroid gland with a heterogeneous echostructure

All routine laboratory investigations, including CBC; ionogram; renal, hepatic, and lipid profiles; viral serologies (hepatitis B, hepatitis C, syphilis, cytomegalovirus, and HIV); and fasting blood glucose, were within normal limits. The urine toxicology screen was also negative. Cerebral MRI and EEG findings were normal. Thyroid ultrasound showed a large hypervascular goiter without identification of a suspicious nodule, findings compatible with Graves’ disease (Figure 2).

The biological and ultrasound findings were consistent with a diagnosis of Graves’ disease. From a psychiatric perspective, the clinical presentation fulfilled the DSM-5 criteria for a manic episode due to another medical condition, namely hyperthyroidism. Several clinical arguments supported this diagnosis: the absence of any personal history of mood disorders; the temporal relationship between the onset of Graves’ disease and the emergence of psychiatric symptoms (the patient reported the onset of exophthalmos six months before admission); and the presence of typical manic symptoms, including insomnia without fatigue, logorrhea, disinhibition, grandiose ideation, excessive spending, clastic behavior, and heteroaggression.

An endocrinology opinion was requested, and the patient was started on antithyroid treatment with methimazole to control hyperthyroidism associated with Graves’ disease. Propranolol was also initiated to control cardiac symptoms related to hyperthyroidism. Methimazole was administered orally at 10 mg three times daily (morning, midday, and evening), for a total daily dose of 30 mg. Propranolol was administered orally at 40 mg twice daily (morning and evening), for a total daily dose of 80 mg.

The diagnosis of hyperthyroidism was established after 14 days of hospitalization. Methimazole 30 mg/day and propranolol 80 mg/day were initiated on day 15. Psychotropic medications (olanzapine 15 mg/day, carbamazepine 400 mg/day, chlorpromazine 200 mg/day, and alprazolam) had been started on admission (day 1) to control acute psychiatric symptoms. After initiation of methimazole and propranolol, olanzapine 15 mg/day and carbamazepine 400 mg/day were continued unchanged, while chlorpromazine and alprazolam were progressively tapered and discontinued following improvement in sleep.

The clinical course under treatment was favorable, with improvement in both psychiatric and somatic symptoms (Table 2). No notable adverse effects were observed with methimazole, propranolol, olanzapine, chlorpromazine, carbamazepine, or alprazolam.

**Table 2:** Clinical course, investigations, and therapeutic management

Time point	Event	Details
Day -7 (before admission)	Onset of symptoms	Insomnia without fatigue, logorrhea, disinhibition, and expression of megalomaniac ideas
Day 0	Hospitalization	Onset of clastic crises and hetero-aggressive behavior toward close relatives, leading to hospital admission. Neuroleptic treatment was initiated on the same day.
Day 2	Laboratory workup and electrocardiogram	Biological workup and electrocardiogram performed
Day 7	Cervical ultrasound	Cervical ultrasound performed
Day 14	Diagnosis established	Diagnosis of Graves’ disease established based on the results of the biological workup, electrocardiogram, and cervical ultrasound
Day 15	Specific treatment initiated	Introduction of methimazole and propranolol
Day 44	Marked clinical improvement	Stabilization of the psychiatric condition and progressive regression of symptoms observed

The patient was discharged after 45 days of hospitalization. At discharge, her condition had significantly improved. No biological assessment was performed at discharge. Chlorpromazine and alprazolam had been discontinued during hospitalization. The following medications were continued at discharge: methimazole 30 mg/day (10 mg three times daily); propranolol 80 mg/day (40 mg in the morning and 40 mg in the evening); olanzapine 15 mg/day (at night); and carbamazepine 400 mg/day (in the morning).

A follow-up thyroid assessment was performed during an endocrinology consultation three months after discharge, while the patient was on methimazole 30 mg/day. The control biological assessment revealed significant improvement, with normalization of thyroid function tests: TSH at 2.5 mIU/L (reference: 0.4-4.0 mIU/L), FT4 at 15 pmol/L (reference: 9-22 pmol/L), anti-TSH receptor antibodies at 15 (previously >50), and anti-TPO antibodies decreased to 30 IU/mL (previously 396.61 IU/mL). This biological normalization was accompanied by complete remission of psychiatric symptoms, supporting the diagnosis of secondary mania induced by hyperthyroidism (Table 3).

**Table 3:** Follow-up laboratory findings after three months of methimazole 30 mg/day FT4, free thyroxine; TPO, thyroid peroxidase; TSH, thyroid-stimulating hormone

Parameter	Patient value	Reference range	Interpretation
TSH	2.5 mIU/L	0.4-4.0 mIU/L	Normal
Thyroxine (FT4)	15 pmol/L	9-22 pmol/L	Normal
Anti-TSH receptor antibodies	15	Negative	Decreased
Anti-TPO antibodies	30 IU/mL	<34 IU/mL	Decreased

The patient underwent regular psychiatric follow-up: the first appointment was one week after discharge, then at one month, two months, and subsequently every three months until April 2026 (Table 4).

**Table 4:** Post-hospital follow-up

Timing after discharge	Type of follow-up	Details
One week after discharge	Psychiatric	First outpatient psychiatric consultation
One month after discharge	Psychiatric	Follow-up psychiatric consultation
Two months after discharge	Psychiatric	Follow-up psychiatric consultation
Three months after discharge	Endocrinological	Specialized endocrinology consultation. No information was available on the frequency of subsequent endocrinology follow-up visits.
Every three months thereafter	Psychiatric	Regular psychiatric follow-up until April 2026

At the last follow-up in April 2026 (18 months after treatment initiation), methimazole was continued as the sole medication at a maintenance dose of 5 mg once daily, as the patient remained clinically and biochemically euthyroid. All psychotropic medications (olanzapine, carbamazepine, chlorpromazine, and alprazolam) had been gradually tapered and discontinued within the first six months following complete psychiatric remission. Propranolol had also been discontinued after normalization of cardiovascular symptoms.

The last follow-up visit in April 2026, conducted at our institution 18 months after treatment initiation (November 2024), showed improvement across all domains: clinical, biological, and psychiatric. Thyroid function tests were within normal limits (TSH and FT4 within reference ranges), and the patient remained clinically euthyroid. Psychiatrically, she was asymptomatic, with complete remission of mood symptoms. She continued maintenance methimazole therapy with good tolerance.

## Discussion

Hyperthyroidism, especially in Graves’ disease, can affect the brain and provoke disorders such as mania, psychosis, or anxiety [9]. In the study by Goyal et al. [11], conducted in a tertiary care hospital in India, the study group comprised 40 consecutive drug-naïve patients with first-episode mania (ICD, 10th Revision), matched with 40 healthy controls. The prevalence of thyroid disorders in this study group was 12.5%, comprising 7.5% cases of overt hyperthyroidism and 5% cases of subclinical hyperthyroidism. These results are consistent with other studies indicating a prevalence of hyperthyroidism in bipolar patients ranging from 2.5% to 32% [12,13].

Recent studies show that the thyroid plays an important role in mood disorders. Hypothyroidism may favor depression, while excess thyroid hormones increase the risk of mania. Thyroid hormones act on β-adrenergic receptors and catecholamines (notably norepinephrine), leading to hyperstimulation of the central nervous system [14]. Moreover, the hyperadrenergic state induced by hyperthyroidism may disrupt adrenergic pathways connecting the frontal lobe to the locus coeruleus, which are involved in the regulation of attention and arousal. These mechanisms may explain psychiatric manifestations such as mania or psychosis in thyrotoxic states [14,15].

Several clinical cases reported in the literature describe manic or hypomanic episodes revealing hyperthyroidism, notably in the context of Graves’ disease (Table 5). Bennett et al. [16] described the case of a young woman without psychiatric history who presented with hypomania and paranoia as initial manifestations of Graves’ disease; after initiation of treatment (carbimazole and propranolol), symptoms regressed without the use of antipsychotics.

**Table 5:** Clinical cases of manic episodes revealing Graves’ disease

Authors	Year	Sex and age	Medical history	Clinical presentation	Treatment	Outcome
Bennett et al. [16]	2021	Female/32 years	None	Hypomania and paranoia as the first manifestations of Graves’ disease	Carbimazole + propranolol (without antipsychotics)	Full remission
Asif et al. [17]	2022	Male/37 years	Known Graves’ disease (poor compliance)	Agitation, hyperactivity, paranoia, and signs of thyrotoxicosis	Methimazole 30 mg, propranolol 100 mg, hydrocortisone 100 mg	Full remission
Spiegel et al. [18]	2025	Female/adult	Known Graves’ disease (poor compliance)	Manic episode: elevated mood, tachyphemia, grandiose and persecutory ideation	Methimazole + antipsychotics	Remission
Ravishankar et al. [19]	2024	Male/adult	None	Excessive spending, agitation, weight loss, tachycardia (thyroid storm in Graves’ disease)	Carbimazole/propylthiouracil + beta-blocker + antipsychotics	Favorable outcome
Our case	2024	Female/31 years	None	First manic episode with psychotic features: logorrhea, delusions of filiation/possession, insomnia	Methimazole 30 mg + propranolol 80 mg; olanzapine 15 mg + carbamazepine 400 mg + chlorpromazine 200 mg	Full remission

Another case published in 2022 [17] describes a 37-year-old man with a history of Graves’ disease and poor therapeutic adherence who presented with agitation, hyperexcitability, hyperactivity, and paranoia with symptoms of thyrotoxicosis requiring hospitalization. Treatment with methimazole (30 mg), propranolol (100 mg), and hydrocortisone (100 mg) was initiated, leading to the resolution of symptoms.

Spiegel et al. [18] described a patient presenting with a manic episode characterized by an elevated mood, hyperactivity, tachyphrenia with flight of ideas, and delusional ideas of grandeur and persecution. According to relatives, these symptoms had a recent onset, evolving for approximately 24 hours before admission. This presentation occurred in the context of Graves’ disease with poor adherence to methimazole treatment.

Ravishankar et al. [19] described a case of a man without psychiatric history who presented with manic symptoms such as excessive spending, overfamiliar behavior, and agitation associated with physical signs of thyrotoxicosis, notably weight loss and tachycardia. He was diagnosed with a thyroid storm occurring in the context of Graves’ disease.

Muhali et al. [20] report the case of a 22-year-old patient presenting with acute psychosis (visual and auditory hallucinations and psychomotor agitation), refractory to 18 weeks of maximal medical treatment (carbimazole + antipsychotics), and secondary to undiagnosed Graves’ disease for two years. The psychosis completely resolved within two weeks after total thyroidectomy, highlighting the importance of routine thyroid function testing in all cases of acute psychosis and the need to escalate to surgery in cases of medical refractoriness. This case also underscores the risk of delayed diagnosis due to psychiatric anchoring bias in resource-limited settings.

The arguments in favor of secondary mania are as follows: the patient has no personal history of depressive, manic, or hypomanic episodes. She reported the onset of exophthalmos approximately six months before admission. Psychiatric symptoms, including insomnia, logorrhea, and hyperactivity, appeared during the hyperthyroid phase, consistent with an organic cause. The clinical presentation included typical manic symptoms such as insomnia without fatigue, hyperactivity, logorrhea, disinhibition, grandiose ideas, and irritability. Biological abnormalities confirmed hyperthyroidism, with suppressed TSH and elevated FT4 levels. Thyroid ultrasound findings, as well as immunological testing, including anti-TSH receptor antibodies, were consistent with Graves’ disease. Furthermore, the favorable response to antithyroid treatment supports the causal role of hyperthyroidism in triggering the manic presentation. The resolution of symptoms after achieving euthyroid status without relapse, as well as the absence of subsequent depressive episodes, argues against a diagnosis of bipolar I disorder [8].

Methimazole belongs to the thionamide class (also called thioureylenes), specifically the imidazole subgroup with a five-membered ring structure, in contrast to propylthiouracil, which belongs to the thiouracil group with a six-membered ring. Thionamides inhibit thyroid hormone production by blocking iodide organification and coupling of iodotyrosine residues within thyroglobulin, essential steps catalyzed by TPO. This inhibition prevents the synthesis of thyroid hormones T3 and T4 in the thyroid gland [21].

Propranolol is a nonselective beta-blocker that blocks β1 and β2 adrenergic receptors, thereby inhibiting the sympathetic effects of catecholamines and reducing tachycardia, palpitations, and tremors associated with hyperthyroidism [22]. Hyperthyroidism causes cardiovascular manifestations such as tachycardia and palpitations. It also leads to increased cardiac output with decreased vascular resistance. Patients may present with reduced exercise tolerance and, in some cases, heart failure. In certain cases, especially in Graves’ disease, rhythm disorders such as atrial fibrillation may be observed [23].

Olanzapine (an atypical antipsychotic) primarily acts by blocking dopamine D2 and serotonin 5-HT2A receptors, which helps reduce both positive and negative symptoms of psychosis while limiting extrapyramidal side effects. It also has affinity for H1, muscarinic, and α1-adrenergic receptors, which explains side effects such as sedation, weight gain, anticholinergic effects, and orthostatic hypotension [24].

Carbamazepine (a mood stabilizer and anticonvulsant) blocks voltage-gated sodium channels by stabilizing their inactive state, thereby reducing neuronal excitability and preventing repetitive discharges. Its effect is frequency- and voltage-dependent, being more pronounced during high neuronal activity. It also reduces synaptic transmission by inhibiting glutamate release and enhancing gamma-aminobutyric acid (GABA) activity, contributing to its anticonvulsant effect [25].

Alprazolam (a benzodiazepine) is a positive allosteric modulator of GABA-A receptors that enhances the effect of GABA by increasing chloride channel opening. This leads to neuronal hyperpolarization and reduced excitability, producing anxiolytic, sedative, hypnotic, and anticonvulsant effects. The α1 subunit is mainly responsible for sedation, while α2 and α3 subunits are involved in anxiolytic and muscle relaxant effects [26].

Chlorpromazine (a typical antipsychotic of the phenothiazine class) primarily acts by blocking dopamine D2 receptors, which reduces positive psychotic symptoms. Its blockade of other receptors explains side effects, including extrapyramidal symptoms (nigrostriatal pathway), orthostatic hypotension (α1), anticholinergic effects (M1), and sedation/antiemetic effects (H1, D2, and M1). It is also metabolized in the liver by CYP2D6, CYP1A2, and CYP3A4 enzymes [27].

Methimazole is indicated in Graves’ disease with hyperthyroidism; toxic multinodular goiter when surgery or radioactive iodine is not appropriate; preparation for thyroidectomy or radioactive iodine therapy; and off-label use in thyroid storm. The recommended dosage in adults is 15 mg/day for mild hyperthyroidism, 30-40 mg/day for moderate hyperthyroidism, and up to 60 mg/day for severe hyperthyroidism, with a maintenance dose of 5-15 mg/day for 12-18 months. In children, the dose is 0.4-0.7 mg/kg/day (maximum 30 mg/day) for one to two years. The daily dose is usually divided into three doses at eight-hour intervals. The main contraindications are hypersensitivity to methimazole, breastfeeding, and pregnancy, where propylthiouracil is preferred in the first trimester due to the risk of embryopathy. Precautions include hepatic monitoring due to the risk of hepatic adverse effects, CBC monitoring for rare but serious agranulocytosis, vigilance for ANCA-associated vasculitis and lupus-like syndrome, and caution at doses above 40 mg/day and in patients over 40 years of age. Methimazole can be used in adults of all ages, in children and adolescents at adjusted pediatric doses, and in newborns and infants under strict supervision due to the risk of fetal goiter and cretinism via transplacental passage [28].

However, the improvement in psychiatric symptoms should be interpreted with caution. In this case, the patient simultaneously received multiple psychotropic treatments, including olanzapine, carbamazepine, chlorpromazine, and alprazolam. Therefore, it is not possible to attribute the clinical improvement exclusively to methimazole. This represents an important limitation in the interpretation of the therapeutic response.

This clinical case highlights the importance of systematic thyroid screening in patients presenting with a first episode of mania, particularly when the onset is abrupt and accompanied by somatic signs suggestive of hyperthyroidism. Future research should evaluate the prevalence of thyroid dysfunction in first-episode mania and optimize the duration of psychiatric treatment in secondary mania. Brain imaging studies could help clarify cerebral changes induced by thyrotoxicosis.

## Conclusions

This case shows that a manic episode can be caused by hyperthyroidism, particularly in Graves’ disease. It is therefore essential to perform a thorough clinical examination and appropriate complementary investigations (thyroid function testing is important in cases of manic episodes) to exclude organic causes before concluding a primary psychiatric disorder.
